# Emerging trends and persistent challenges in the management of adult syphilis

**DOI:** 10.1186/s12879-015-1028-3

**Published:** 2015-08-19

**Authors:** Susan Tuddenham, Khalil G. Ghanem

**Affiliations:** Johns Hopkins University School of Medicine, 5200 Eastern Ave, MFL Center Tower #378, Baltimore, MD 21224 USA; Division of Infectious Diseases, 1830 E. Monument Street, Room 442, Baltimore, MD 21287 USA

**Keywords:** Syphilis, *Treponema pallidum*, Neurosyphilis, Therapy, Diagnostics

## Abstract

There are an estimated 10.6 million incident cases of syphilis worldwide each year. We highlight some persistent challenges and emerging trends in the clinical management of syphilis with a particular focus on therapy, serology, diagnostics, and prevention. Decades after the introduction of penicillin, the optimal management of early syphilis continues to be a controversial topic, particularly in the setting of HIV co-infection. Similarly, the need for routine lumbar puncture in HIV co-infected asymptomatic persons is an unanswered question. Despite advances in both automation and point-of-care diagnostics, we continue to rely on indirect measures of disease activity to manage this infection. As syphilis rates in some populations continue to rise, novel and effective prevention strategies are needed.

## Review

Worldwide, there are an estimated 10.6 million incident cases of syphilis each year [1]. Recent increases in rates of syphilis have been observed in high-income countries among men who have sex with men (MSM), many of whom are HIV co-infected [2–4]. While strict testing and treatment guidelines have significantly decreased morbidity and mortality of congenital syphilis in high-income countries, syphilis infection in pregnancy remains a significant cause of adverse pregnancy outcomes in many others [5, 6]. We were invited by the editors of BMC Infectious Diseases to contribute a review on current challenges in the management of syphilis. In this review,we highlight some emerging trends and persistent challenges in the clinical management of syphilis with a particular focus on therapy, serology, diagnostics, and prevention.

### Therapy

Decades after the introduction of penicillin, the optimal management of early syphilis continues to be a controversial topic, particularly in the setting of HIV co-infection. Currently, most major clinical guidelines recommend similar treatment regimens for the various stages of syphilis (Table 1). Penicillin continues to be the drug of choice to treat all stages of syphilis in all populations with tetracyclines and cephalosporins acceptable alternate agents for some stages in non-pregnant persons [7]. In general, the emergence of drug resistance has precluded recommending macrolides for the routine treatment of early syphilis [8, 9].

**Table 1:** Treatment of syphilis: US, UK and European recommendations

Stage	US CDC- recommended regimens [11]	UK recommended regimens [10]	European recommended regimens [12]	US alternative regimens [11]	UK alternative regimens [10]	European alterative regimens [12]
Early syphilis (primary, secondary, early latent of <1-2 years’ duration	Benzathine penicillin G 2.4 million units IM single dose	Benzathine penicillin G 2.4 million units IM single dose or Procaine penicillin G 600 000 units IM q24h for 10 days	Benzathine penicillin G 2.4 million units IM single dose	Doxycycline 100 mg or PO q12h for 14 days Ceftriaxone 1-2 g IM q24h for 10 days	Doxycycline 100 mg or PO q12h for 14 days Erythromycin 500 mg or PO q6h for 14 days Ceftriaxone 500 mg IM or daily for 10 days Amoxicillin 500 mg PO q6h plus Probenecid 500 mg or po q6h for 14 days	Procaine penicillin 600 000 units IM or daily for 10-14 days Doxycycline 200 mg or daily orally for 14 days Ceftriaxone 500 mg-1 g IM or IV daily for 10 days Azithromycin 2 g orally single dose
Late syphilis (latent syphilis of >1-2 years’ duration, cardiovascular, gummatous syphilis	Benzathine penicillin G 2.4 million units IM weekly for 3 weeks	Benzathine penicillin G 2.4 million units IM or weekly for 3 weeks Procaine penicillin 600 000 units IM q24h for 17 days	Benzathine penicillin G 2.4 million units IM weekly for 3 dose	Doxycycline 100 mg PO q12h for 28 days	Doxycycline 100 mg or po q12h for 28 days Amoxicillin 2 g PO q8h plus probenecid 500 mg or po q6h for 28 days	Procaine penicillin 600 000 units IM or daily during 17-21 days Doxycycline 200 mg PO daily during 21-28 days
Neurosyphilis	Aqueous crystalline penicillin G 3-4 million units IV q4h for 10-14 days	Procaine penicillin 1.8-2.4 million units IM q24h plus probenecid 500 mg or po q6h for 17 days Benzyl penicillin 3-4 million units IV q4h for 17 days	Benzyl penicillin 3-4 million units IV q4h for 10-14 days	Ceftriaxone 2 g IM or IV q24h for 10-14 days	Doxycycline 200 mg po q12h for 28 days Amoxicillin 2 g PO q8h plus probenecid 500 mg or PO q6h for 28 days Ceftriaxone 2 g IM or IV q24h for 10-14 days	Ceftriaxone 1-2 g or IV daily during 10-14 days Procaine penicillin 1.2-2.4 million units IM daily and Probenecid 500 mg po q6h for 10-14days

IM: intramuscular, IV: intravenous, PO: by mouth, q4h: every 4 h, q6h: every 6 h, q12h: every 12 h, q24h: every 24 h, q48h: every 48 h, mg: milligrams, g: grams

Although most guidelines recommend similar treatment regimens for HIV-infected and uninfected persons with syphilis, debate has centered on whether enhanced therapy provides improved clinical or serological outcomes for HIV-co-infected persons [10–12]. The predilection for the development of early symptomatic neurosyphilis among HIV-infected persons [13, 14] and data showing that a single dose of 2.4 MU of benzathine penicillin G (BPG) may be insufficient to clear *Treponema pallidum* from the CSF of HIV-infected persons with early syphilis [15] have fueled this debate. Particular interest has focused on enhanced therapy using three intramuscular doses of 2.4 MU of BPG instead of one. In the pre-HIV era, three doses of 2.4 MU BPG were recommended as an alternate treatment regimen for neurosyphilis [16]. In the early 1980’s its use was abandoned, not because of lack of efficacy, but because it did not achieve consistent treponemicidal penicillin concentrations in the CSF [17]. Results of retrospective studies comparing one versus multiple doses of BPG were inconsistent [18]. The only published randomized controlled trial used a non-standard regimen for neurosyphilis. It was also underpowered to address the question in HIV-infected persons [19]. Recently, two studies tried to address the question. The first, a prospective multicenter Taiwanese study comparing 1 vs. 3 doses of BPG in HIV-infected patients, found no statistically significant differences in serological responses at 6 and 12 months between the two groups in the per protocol analysis, though a carry-forward analysis did suggest improved serological outcomes favoring the three dose group [20]. The second study, a retrospective review of 478 cases of early syphilis in HIV-infected patients followed in the US Military HIV Natural History Study, found no statistically significant difference in serologic response at 13 months in those receiving one dose of BPG versus those receiving ≥ 2 doses of BPG [21]. Neither study assessed clinical outcomes (*e.g*. development of early neurosyphilis) in a systematic way. As such, the question of enhanced therapy remains unanswered. Despite the fact that US guidelines are clear and unambiguous about single dose penicillin to treat early syphilis among HIV infected persons, many clinicians continue to use enhanced therapy [22].

### Serologies

*T. pallidum* cannot be cultured in the laboratory and the performance characteristics of direct molecular diagnostics are limited [23–27]. Consequently, diagnosis and monitoring of treatment responses depend largely on serological testing. (The use of darkfield microscopy for the diagnosis of early syphilis using specimens from genital lesions, and the use of PCR testing on these lesions, which is even more sensitive, are rarely performed outside of specialized settings). The reliance on these indirect tests that may not necessarily reflect underlying disease activity has generated significant challenges in the management of syphilis [28].

#### The serofast state

Patients who fail to achieve a 4-fold (*i.e*. two-dilution) decline in nontreponemal [*i.e*. Venereal Diseases Research Laboratory (VDRL) or Rapid Plasma Reagin (RPR)] antibody titers, or those who have adequate serologic decline but do not completely revert from positive to negative have been referred to as ‘serofast’ [7]. The definition is broad and it is unclear if both categories should be grouped together. In this section, we define ‘serofast’ as those who do not achieve a four-fold decline in titers following stage-appropriate therapy [29].

The clinical importance of the serofast state was clearly documented in the pre-antibiotic era. During that time, 30 % of patients with early syphilis who were ‘Wasserman fast’ (the Wasserman test was a nontreponemal predecessor to the RPR and VDRL) developed late neurologic complications, though this was not seen in latent syphilis [30]. In the modern era, the optimal approach to managing a patient who fails to achieve a four-fold decline in nontreponemal antibody titers (and who was not reinfected) is not known. In one study, among 13 HIV-infected patients with serologic evidence of syphilis whose serologic titers failed to decline adequately a median of 287 days after therapy, 4 (31 %) were found to have CSF abnormalities consistent with asymptomatic neurosyphilis [31]. However another study did not find an increased frequency of CSF abnormalities in serofast HIV positive patients, but different definitions of serofast serologies were used [32].

In some cases, a longer duration of follow-up is necessary to document four-fold titer declines [33]. Indeed, persons with late syphilis [34] and those with HIV not on antiretroviral therapy exhibit slower serological responses [32]. Persons with a lower initial RPR may be more likely to remain serofast [29].

The short-term outcomes of treating patients who are serofast have been described. One study demonstrated that only a modest proportion (27 %) of HIV uninfected serofast patients with early syphilis achieved a serologic response 6 months after retreatment with one dose of 2.4MU BPG. There was no comparison group, so it is difficult to conclude that this response was solely attributable to retreatment [35]. Indeed, a study of patients with late latent syphilis found similar serological responses between treated and untreated serofast patients at the end of follow-up [33]. The more vexing problem lies beyond the short-term serological responses: What are the long-term clinical consequences of being serofast? At this time, the question remains unanswered. Nonetheless, current CDC guidelines recommend additional clinical and serological follow-up in serofast patients and retreatment if follow-up cannot be assured. Because treatment failure can be the result of unrecognized CNS infection, CDC guidelines further recommend that CSF examination be considered in these patients, if reinfection has been ruled out [11].

### Diagnostics

Fully automated assays that detect treponemal antibodies and novel point-of-care (POC) diagnostics have significantly impacted the field of syphilis diagnostics.

#### The reverse sequence algorithm

Increased availability of automated testing has recently led many laboratories to switch to a reverse sequence algorithm (RSA) in which a treponemal test is conducted first, and if positive, followed by a confirmatory nontreponemal test [36]. Data on whether the RSA is cost effective in low prevalence settings are mixed, with one study from Canada finding that it was cost effective as compared with the traditional algorithm, and one study from the US finding that it was less cost effective [37, 38]. It does appear that the RSA may facilitate the identification of latent syphilis cases, though it may lead to more false positives as well [39–41].

The use of the RSA rather than the traditional nontreponemal-first algorithm does result in the identification of a number of patients with serodiscordant results (*i.e*. a positive treponemal test and a negative nontreponemal test) [36]. This can occur in the setting of 1) a false positive result, 2) early primary syphilis, 3) the prozone phenomenon, 4) adequately treated past syphilis or 5) untreated syphilis of long duration, as nontreponemal test titers may decline over time even in the absence of treatment. A second treponemal test (preferably based on different antigens than the original test) should be conducted to rule out the first possibility. Persons with a confirmed positive treponemal test and a negative nontreponemal test with a history of syphilis treatment will require no further management unless there is concern for reinfection [36].

Persons with serodiscordant serologies (*i.e*. confirmed positive treponemal and persistently negative non treponemal tests) and no history of syphilis treatment present several important clinical and public health questions, particularly since they would likely be missed by the traditional algorithm: What is their risk of transmitting syphilis sexually? What is their risk of transmitting syphilis vertically? What is their risk of progression to tertiary syphilis? Based on studies from the pre antibiotic era, the risk of sexual transmission in patients with late syphilis is low [42]. Vertical transmission of syphilis has been documented at around 10 % in mothers with late latent disease [43], while one study from 1922 reported that nearly 50 % of infants born to seronegative mothers had probable or definitive congenital syphilis [44]. However, the risk of vertical transmission in serodiscordant women in the antibiotic era when patients are often routinely exposed to beta lactam antibiotics, tetracyclines and macrolides for reasons other than syphilis treatment, combined with the use of significantly more sensitive modern nontreponemal tests is unknown. A recent retrospective review suggests that it is low [45]. The risk of progression to tertiary syphilis in serodiscordant patients in the modern antibiotic era is unknown and ethical and logistical challenges make it unlikely that a study to address this issue will be forthcoming. Two retrospective studies (Wohrl and Tuddenham) suggest that the risk of neurosyphilis at the time of discordant test results is low. The risk of ophthalmic syphilis may be higher, but the lack of objective measures for this diagnosis makes drawing conclusions difficult [46, 47].

In general, persons with serodiscordant results and no history of treatment should be assessed clinically, and treated appropriately if signs or symptoms of active syphilis are noted. If none are found, they should be treated for syphilis of unknown duration with three doses of 2.4MU of BPG weekly [48].

#### Point-of-care tests

Point of care (POC) tests are both simple and capable of providing rapid results to guide clinical decisions during the same encounter [49]. In recent years, a number of rapid POC tests, which can deployed in a variety of settings and provide results without specialized laboratory equipment, have been developed for syphilis [50–53]. These include treponemal tests, combination treponemal and nontreponemal tests, and multiplex tests that simultaneously test for syphilis antibodies and antibodies to other infections such as HIV and hepatitis C [50, 53–55]. The majority of these tests rely on lateral flow technology. Several are commercially available internationally. There is currently one which is FDA approved for use in the United States [52]. Recently, this test was granted a Clinical Laboratory Improvement Amendment (CLIA) waiver that will enable its use outside of traditional laboratory settings [56].

Efforts are underway to miniaturize current laboratory-based technologies to enable rapid enzyme linked immunosorbent assay (ELISA) tests to be run in the field. A recent paper reports on the development and deployment of a smartphone attachment, or “dongle,” which enables a triplex ELISA test that provides results for HIV, treponemal, and non-treponemal antibodies [57].

POC syphilis tests show considerable promise, however they do have some limitations. Most tests are based on treponemal antibodies and therefore cannot distinguish between new and previously treated infections. Combination treponemal and non-treponemal POC tests, which may offer more information, do not provide a non-treponemal titer with which to follow response to treatment. Therefore, in settings where laboratory access is available, POC tests may have less utility for general use. In settings where access to laboratory facilities is limited and where prevalence may be high, POC tests may play an integral role in the diagnosis of syphilis. An example is antenatal clinics, where the consequences of overtreatment may be relatively small, but the consequences of undertreatment may be catastrophic. A number of studies, including one large study in which POC’s were introduced in project sites in six countries demonstrated the feasibility of utilizing POC’s for antenatal screening in a variety of rural and urban settings, and significantly increased the proportion of pregnant women screened and treated for syphilis [58]. Mathematical modeling suggests that use of POC tests for antenatal syphilis screening in low resource settings is not only effective but cost effective as well [59].

Point of care syphilis testing in low resource settings may soon expand to include other specimens. Ho et al. optimized the “Syphicheck” POC test made by Qualpro Diagnostics, Goa, India, which was designed for use on blood to be used on CSF, and obtained results similar to those obtained with a CSF VDRL [60]. This could have important implications for the diagnosis of neurosyphilis in settings without extensive laboratory capabilities.

#### Neurosyphilis diagnostics

The diagnosis of neurosyphilis is challenging because there is no gold-standard diagnostic test. It requires a CSF examination, but the indications to perform this procedure remain an issue of debate. CDC guidelines currently recommend that a CSF examination be performed in 1) persons with neurologic signs or symptoms 2) persons diagnosed with tertiary syphilis (*e.g*. cardiovascular syphilis or late benign syphilis) and 3) asymptomatic persons whose serologic titers do not decline appropriately following recommended therapy [11]. This last recommendation remains the source of some controversy (as detailed above). The most controversial question is the need for a CSF examination in asymptomatic HIV-infected persons with CD4 cell counts ≤ 350 cells/mm^3^ or RPR titers ≥ 1:32. The last two criteria were noted to increase the probability of symptomatic and asymptomatic neurosyphilis in this population [31, 61]. At this time, data are lacking regarding the benefits of CSF examination in asymptomatic HIV-infected persons. Consequently, none of the major guidelines have adopted a formal recommendation for this population.

Once the CSF examination is obtained, interpreting the results may present further challenges. The CSF VDRL is the most specific test but it is only about 50 % sensitive. The CSF RPR is less sensitive than the CSF VDRL and should not be used for diagnosis of neurosyphilis [62]. CSF treponemal tests are more sensitive but less specific and a negative CSF treponemal test in the setting of a moderate to high pre-test probability of neurosyphilis does not rule out the diagnosis (reviewed by Harding) [63, 64]. The CSF pleocytosis in syphilis is lymphocyte predominant, and a cutoff of greater than or equal to 5 cells/ml has been standard; however this may have less specificity in HIV-infected persons [65].

Novel neurosyphilis diagnostics are being sought. Several papers have evaluated CSF CXCL-13, a B-cell chemoattractant, as a diagnostic test for neurosyphilis [66–68]. Marra et al. examined serum and CSF concentrations of CXCL-13 in 199 HIV-positive patients with early syphilis who were referred because of concern for neurosyphilis. The CSF concentration of CXCL-13 as well as a CSF: serum CXCL-13 ratio of >1 performed reasonably well compared to the CSF VDRL [66].

### Public health interventions

Despite effective treatment and promising new diagnostics, syphilis rates have continued to increase in MSM, most significantly in HIV-infected MSM. A number of factors may be contributing to this, among them the practice of serosorting and seropositioning, often in place of conventional risk reduction preventive strategies such as condom use. In this setting the increased rates of unprotected anal intercourse, particularly in HIV positive men, may be contributing to transmission of syphilis and other STIs [69–71]. Given continued risk behaviors and increasing incidence of syphilis, new prevention strategies are needed. Some possible approaches include frequent screening, novel approaches to case finding, and consideration of chemoprophylaxis.

#### Frequent screening

Syphilis infection and reinfection rates in MSM and HIV infected MSM are high, and identifying and treating asymptomatic early infections may be important in breaking the chain of transmission [72, 73]. Annual screening of these populations has been shown to identify a significant number of asymptomatic infections [74]. However, other studies suggest that implementing more frequent screening in selected populations may have added benefits. One study in which routine syphilis serology was included with every blood test performed as part of HIV monitoring among HIV positive MSM attending a clinic in Australia found a marked increase in identification of early syphilis cases [75]. Furthermore, a study using mathematical models suggests that increasing the frequency of screening in certain high-risk target groups (such as MSM who have large numbers of partners or engage in group sex) to every 3 months may be critical to decreasing syphilis incidence [76]. Another model suggests that increased coverage and frequency of screening of HIV positive MSM may be cost effective [77]. Similarly, screening in incarcerated populations may be high yield, [78] and may be cost effective as well [79].

#### Case finding: novel approaches

The internet and mobile devices are increasingly being used to locate sex partners. New approaches that make use of the internet and mobile phone texting technologies to assist in partner notification, contact tracing, education, and testing must constantly evolve to reach at-risk groups [80–82]. Several studies have associated risk behaviors in MSM, including unprotected anal intercourse, with use of novel social networking applications [83, 84]. A recent report described increased syphilis testing with the use of a promotion campaign on the social networking site Grindr, which is often used by MSM to find sexual partners [85].

#### Antibiotic prophylaxis and other approaches

While mass antibiotic treatment, conducted in outbreak situations or high-prevalence settings, has had mixed results in affecting the incidence of syphilis [86, 87], targeted chemoprophylaxis of motivated high-risk individuals may be a reasonable strategy. A recent study demonstrated that over 50 % of MSM surveyed would be willing to take daily pills to decrease their risk of syphilis infection, and over 75 % were willing to take daily medication to decrease syphilis infections in the gay community. Mathematical modeling suggested that chemoprophylactic doxycycline, either widely distributed or targeted to certain high risk MSM, could significantly reduce new cases of syphilis in the MSM community, though cases would rebound once the intervention was stopped [88]. A recent pilot study showed the feasibility of daily doxycycline in a small group of high risk MSM. The study was not powered to show a difference in syphilis incidence, but there was a decrease in acute STIs in the group taking daily doxycycline [89]. This is an intriguing new approach, though larger studies will be required, and several important issues must be carefully weighed before moving forward, such as adherence, impact on the microbiome, emergence of drug resistance, and long-term drug toxicities. Finally, a recent study showed that male circumcision was associated with a decreased risk of incident syphilis in men and women [90].

## Conclusions

Advances in diagnostics may have a significant impact on reducing cases of congenital syphilis in resource-limited settings, where rates of this devastating condition are still very high. Whether these diagnostics can have a meaningful impact on rising rates of syphilis among MSM is unclear. Without a vaccine in sight, novel and effective prevention strategies are sorely needed to combat this infection.
